# Exploring the knowledge, attitudes and practices (KAP) of health care professionals on viral hepatitis notification in Gauteng, South Africa, 2015

**DOI:** 10.1186/s13690-018-0319-8

**Published:** 2018-12-06

**Authors:** Eva D. Mathatha, Jack M. Manamela, Alfred Musekiwa, Nishi Prabdial-Sing

**Affiliations:** 1 School of Health Systems and Public Health, Faculty of Health Sciences, University of Pretoria, Private bag x323, Pretoria, 0007 South Africa; 2 South African Field Epidemiology Training Program, National Institute for Communicable Diseases (NICD), Johannesburg, South Africa; 3 Centre for Vaccines and Immunology, National Institute for Communicable Diseases, Johannesburg, South Africa; 4 Department of Virology, School of Pathology, University of Witwatersrand, Johannesburg, South Africa

**Keywords:** Knowledge, Attitudes, Practices, Health care professionals, Nurses, Doctors, Notification, Viral hepatitis

## Abstract

**Background:**

Data on viral hepatitis in South Africa is scarce. Although viral hepatitis A, B and C are notifiable conditions in South Africa, discrepancies have been noted in the number of viral hepatitis cases notified by the National Department of Health (NDOH) compared with laboratory confirmed cases from the National Institute for Communicable Diseases (NICD). The aim of the study was to assess the knowledge, attitudes and practices of health care professionals on the notification of viral hepatitis A, B and C.

**Methods:**

A descriptive, cross-sectional study on 385 health care professionals was conducted at Charlotte Maxeke Johannesburg Academic and Tshwane District hospitals in Gauteng province, South Africa, between March and May 2015. A pre-tested, structured questionnaire with 21 (6 demographic and 15 knowledge, attitudes, and practice (KAP)) questions was used to collect information from invited participants. A score was assigned to each KAP question and a mean (SD) score was calculated for each section. Data were analyzed using descriptive statistics in STATA version 13.

**Results:**

Of the total 385 respondents, 65% (*n* = 250) were nurses and 35% (*n* = 135) were doctors. The overall mean knowledge score for health care professionals was 2.0 ± 1.6 (mean ± SD) out of a score of 6 regarding viral hepatitis notification. Overall mean scores of practice and attitude towards notification were higher at 2.9 ± 0.4 and 3.3 ± 0.7, out of a score of 4 and 5, respectively. Lack of training, poor knowledge, a complex process and excessive workload were some of the reasons for poor notification of viral hepatitis.

**Conclusions:**

Overall, knowledge on notification of viral hepatitis was poor among health care professionals. Adequate training on viral hepatitis, notification process, roles and responsibilities of health care professionals to notify and the implication of viral hepatitis notifications is recommended to improve reporting rate of notifiable diseases and referrals to increase linkage to care.

**Electronic supplementary material:**

The online version of this article (10.1186/s13690-018-0319-8) contains supplementary material, which is available to authorized users.

## Background

Hepatitis is an inflammatory dysfunction of the liver that can be caused by viruses, drugs, and toxins. There are five types of viral hepatitis (A-E), however, only three are of major public health concern: hepatitis A (HAV), B (HBV), and C (HCV). Together HBV and HCV cause 80% of hepatocellular carcinoma (HCC, [1]), which is the fifth most common cancer in men and seventh in women [2].

In order to meet the goals of the Global Health Sector Strategy (GHSS) to eliminate viral hepatitis by 2030, we need to strengthen surveillance systems to increase diagnoses and treatment [3]. A simple, effective and efficient notifiable diseases surveillance system (NDSS) can benefit health systems with epidemiological data to plan programs for public health interventions and treatment [4]. However, under-reporting of notifiable infectious diseases still remains a major limitation in health care systems worldwide [5, 6]. Over a six-month period in 1993, Rustomjee and Abdool Karim [7] reported that at King Edward VIII hospital, Durban, South Africa, 83% of hospitalized patients with hepatitis B were not reported via the notification system, 6% were incorrectly notified, and no hepatitis B positive cases for hospital outpatients were reported.

Common reasons for under-reporting included lack of time, poor overall knowledge on notifiable conditions, poor knowledge on viral hepatitis tests and interpretation of results among health care professionals, insufficient incentives, and lack of enforced penalty for not reporting [6–8]. A survey conducted in South Africa [9] indicated that the main reasons for under-reporting among doctors was the unavailability and complexity of the notification forms as well as poor feedback to health care professionals.

South Africa has had a routine system for reporting notifiable medical conditions (NMCs) since the late 1970s [4] whereby all health care professionals who come into contact with a patient presenting with one of the prescribed notifiable medical conditions is obliged to notify the condition to their local health care facility which is then escalated from sub-district to national level on the reporting structure framework [10]. There were 33 NMCs in South Africa [4] and the notifiable medical condition surveillance system is being expanded to include more conditions. At the time of the study, a notification form (GW17/5) was to be used at the clinic site. While health care professionals are required to report episodes of acute disease, laboratories in public health facilities in South Africa are required to notify the NDOH based on confirmed positive results [10, 11]. In 2013, inconsistent data on the number of viral hepatitis B and C cases reported to the NDOH and the laboratory confirmed cases at the National Institute for Communicable Diseases (NICD) were observed [12].

The aim of this study was to assess the knowledge, attitudes and practices of health care professionals on the notification of viral hepatitis A, B and C in two hospitals in Gauteng province, South Africa.

## Methods

### Study design

A descriptive, cross-sectional study was conducted between March and May 2015 at Charlotte Maxeke Johannesburg Academic hospital and Tshwane District Hospital in Gauteng, South Africa. Gauteng is the most highly populated province with large referral hospitals where the majority of cases are referred. Cases from other provinces are also referred for treatment and care. The study was approved by the Faculty of Health Science Research Ethics Committee, University of Pretoria (reference number 401/2014). Permission to conduct the research study at the two hospitals was granted by the hospital clinical managers.

### Sample size calculation and sampling plan

Participants of the study were the nurses and doctors working in these hospitals. A sample size of 385 was calculated using Epi Info version 7 with an estimation that 50% of health care professionals had knowledge on notification, while a confidence interval and margin of error was set at 95 and 5%, respectively. A convenience sampling method was used to select study participants. Any health care professional available during data collection was invited to participate until the required sample size was reached.

### Data collection and analysis

A structured questionnaire was developed based on the variables of interest and guided by the study specific objectives. The questionnaire was administered through one-on-one interviews with the healthcare professionals. The questionnaire had 21 questions divided into four sections: 6 questions on demographic information, 6 on evaluating the knowledge of participants on the notifiable hepatitis viruses, signs and symptoms, person responsible for notification and the forms used for notifying, 4 on the practice of the notification process, and the last 5 on the attitude of respondents to notifying, the notification process and the usefulness of notification. Apart from the demographic information, the analysis was conducted on 15 questions on knowledge, attitudes and practices. Each correct/positive answer was given a score of “1” and each wrong/negative answer was given “0”. Data was captured with Epi Info version 7 and the descriptive statistics such as mean, standard deviation and frequencies were generated using STATA version 13. Categorical variables were compared using Chi-square tests and a *p*-value of less than 0.05 was considered statistically significant.

## Results

### Demographics

Of the 385 health care professionals interviewed, 235 (61%) were from Charlotte Maxeke Academic Hospital and 150 (39%) from Tshwane District Hospital. Among these, 65% (*n* = 250) were nurses and 35% (*n* = 135) were doctors. The majority of the respondents were females (74%).The mean (±SD) age of the respondents was 32.8 (±4.8) years.

### Knowledge

**Fig. 1:**
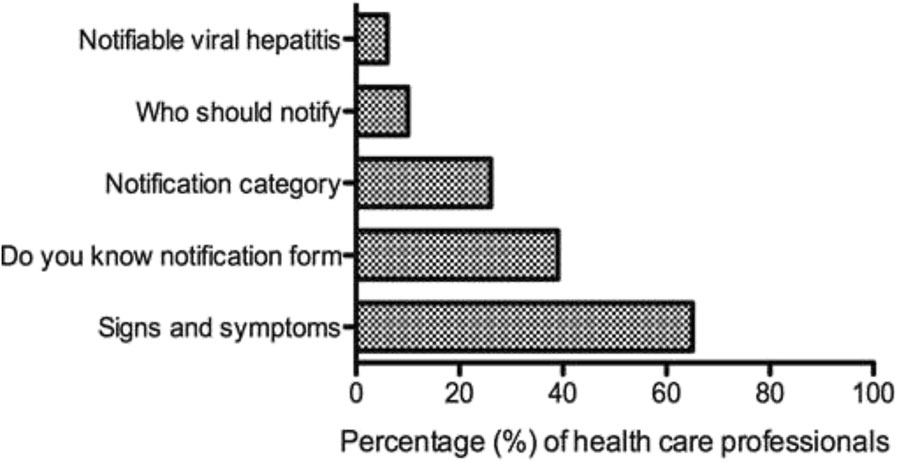
Percentage of health care professionals who correctly answered on knowledge about viral hepatitis notification, Gauteng province, South Africa 2015

**Fig. 2:**
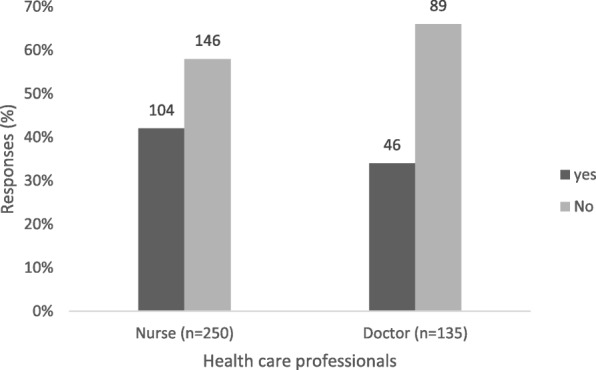
Health care professional’s responses to knowledge of the notification form. Absolute numbers reported on bars, Gauteng province, South Africa 2015

**Fig. 3:**
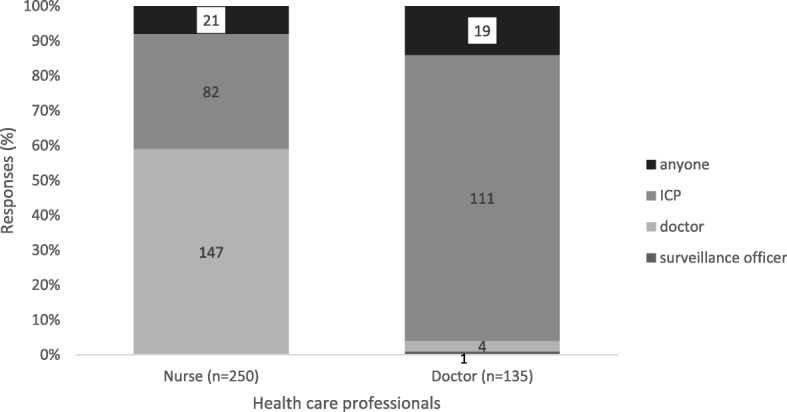
Health care professional’s responses to knowledge on who is responsible to notify. Absolute numbers reported in bars, Gauteng province, South Africa 2015

Sixty-five percent (*n* = 250) of all health care professionals enrolled in the study knew the signs and symptoms of viral hepatitis (Fig. 1). Only 39% (*n* = 150) knew about the notification form (Fig. 1), with 42% (*n* = 104) of the nurses having knowledge about the GW17/5 form compared to 34% (*n* = 46) of the doctors (*p* = 0.148; Fig. 2). On specific knowledge about viral hepatitis notification, 6% (*n* = 16) of nurses and 14% (*n* = 19) of doctors knew that viral hepatitis A, B and C infections are notifiable (Additional file 1: Table S1). The majority (82%, *n* = 205) of nurses were of the view that only hepatitis B is notifiable whilst 50% (*n* = 67) of the doctors thought that only hepatitis C is notifiable (*p* < 0.001, data not shown). On knowledge about who should notify, 59% (*n* = 147) of nurses perceived that doctors should notify, while 82% (*n* = 111) of doctors perceived that the infection control nurse should notify (*p* < 0.001, Fig. 3). Only 8% (*n* = 21) of the nurses versus 14% (*n* = 19) of the doctors knew that any health care professional who first come into contact with the patient should notify the NDOH (*p* = 0.059; Fig. 3).

### Attitudes

**Fig. 4:**
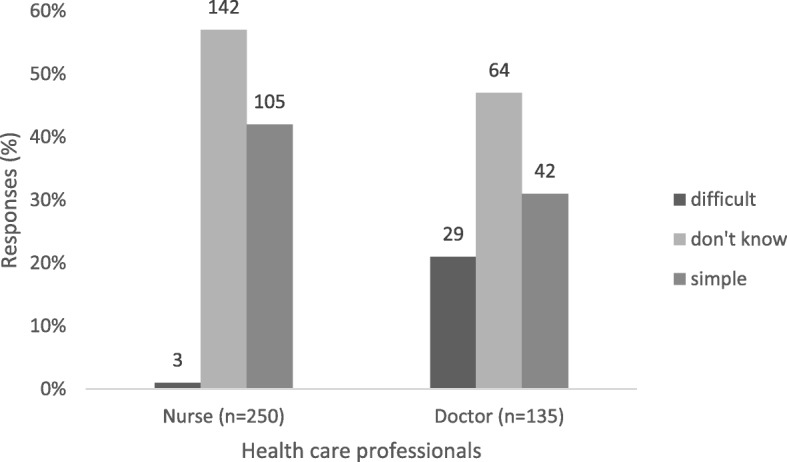
Health care professional’s responses to attitudes on notification process. Absolute numbers reported on bars, Gauteng province, South Africa 2015

The notification process was considered to be difficult by 21% (*n* = 29) of the doctors compared to 1% (*n* = 3) of the nurses (p < 0.001; Fig. 4). More than 90% of nurses (*n* = 232) and doctors (*n* = 128) believed that notification of viral hepatitis is useful (Additional file 1: Table S1).

### Practices

**Fig. 5:**
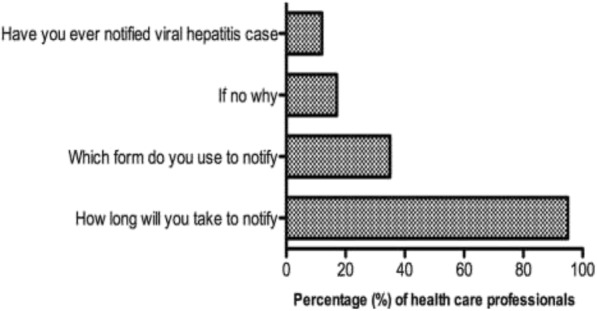
Percentage of health care professional’s responses on practices towards viral hepatitis notification, Gauteng province, South Africa 2015

Thirty-five percent (*n* = 135) of the health care professionals reported that they had used the GW17/5 form to notify. More than 90% of the health care professionals responded that they would notify as soon as possible (Fig. 5). The majority (93%, *n* = 233) of the nurses and doctors (78%, *n* = 105) reported that they had not notified any viral hepatitis case previously (*p* < 0.001, Additional file 2: Figure S1). Nurses (56%, *n* = 139) reported that they had not notified because it is the responsibility of the doctor to notify while 78% (*n* = 105) of the doctors said they had not notified because it is the responsibility of the infection control nurse to notify (*p* < 0.001, Additional file 2: Figure S2).

A breakdown of the percentage scores for each question is provided in Additional file 1: Table S1.

### KAP score

The reliability of the questionnaire was calculated using the Cronbach’s alpha and a value of 0.70 was found, which indicated good reliability. The mean (standard deviation) of knowledge, attitudes and practices were 2.0 (±1.6) out of 6, 3.3 (±0.7) out of 5 and 2.9 (±0.4) out of 4, respectively. The mean (standard deviation) of total score was 8.0(±2) for nurses and 8.8(±2) for doctors, out of 15 (Table 1).

**Table 1:** Mean ± Standard Deviation KAP scores of health care professionals in Gauteng province, South Africa, 2015

Occupation	Knowledge	Attitudes	Practice	Total score
Nurses (*n* = 250)	1.8 ± 1.6	3.3 ± 0.7	2.9 ± 0.4	8.0 ± 2.2
Doctors (*n* = 135)	2.3 ± 1.5	3.3 ± 0.7	3.0 ± 0.5	8.8 ± 2.3
Overall	2.0 ± 1.6	3.3 ± 0.7	2.9 ± 0.4	8.3 ± 2.2
Maximum score	6	4	5	15

## Discussion

The fact that viral hepatitis A, B and C are notifiable diseases was not known among all health care professionals, leading to under-reporting. Poor knowledge (score of 2/6) with regard to viral hepatitis notification among health care professionals was evident. A similar finding was reported among doctors in Taiwan, with an added reason of not knowing which diseases are notifiable [8]. In a previous study conducted at King Edward VIII hospital in KwaZulu-Natal province, South Africa, only 4% of medical doctors had knowledge that hepatitis A is notifiable and only one doctor knew that both hepatitis B and C are notifiable [13]. Similarly, there was a lack of knowledge on NMCs in Spain as doctors notified only severe diseases [14]. Recent studies with similar findings include those carried out in SouthAfrica, on compliance of the health care workers with notifiable disease surveillance system [15].

It was observed that more than half of the nurses and two thirds of the doctors did not know how to complete the notification form or where to obtain the form. These findings are consistent with studies conducted among doctors in Portugal and Nigeria, where it was found that only 10 and 12% of the doctors, respectively, had a good knowledge of the disease notification system and 24% knew where to obtain and how to complete the notification form [16, 17].

The poor score for knowledge among health care professionals observed in the two hospitals was attributed to lack of training on NMCs as the majority of nurses indicated that they had not received training on the disease notification system. Proper training on notification systems has been demonstrated to have a positive impact on health care professionals in northern Nigeria as reporting of notifiable diseases increased from 2.3 to 52% [16].

In the present study, more doctors than nurses indicated that the forms were too complicated to complete. Some of the reasons provided by doctors included excessive workload and the lengthy time that the notification process requires. As early as the 1990’s, the lack of training on the importance of disease notification was found to be one of the reasons for poor compliance among health care professionals [18]. In our study, albeit the poor knowledge and compliance, a high proportion (95%) of health care professionals believed that notification of viral hepatitis is useful, in contrast to the study in Portugal where 68% of health care professionals believed that notification was not useful [16].

The majority of the health care professionals had not notified any case in the present study. The reasons for this could be attributed to the lack of cases to notify at these hospitals or that the responsibility of notification was shifted between doctors and nurses, hence contributing to under-reporting. The most common reason for not notifying among health care professionals in our study was the perception that it is someone else’s responsibility.

Several years ago, reporting rates of medical practitioners and laboratories differed; 75% of cases were reported by laboratories, 20% by medical practitioners and almost 5% by both, with laboratories reporting more quickly than medical practitioners [19, 20]. Recently, Benson et al., [21], reported that the laboratory system reported more cases of three tracer diseases in South Africa (measles, meningococcal meningitis and typhoid) compared to the NDSS, with laboratories scoring higher on system attributes such as completeness, stability and representativeness.

An alert system seems pivotal to not only notify but also engage health care professionals and laboratories to link patients to care. In a study in Japan, an automated alert system improved notification from 46 to 73% and referrals from 16 to 27% in a hospital setting [22]. A study at Osaka City hospital, Japan, Fujii et al., [23] indicated that the use of “Private doctors’ practices” on electronic medical records significantly increased referrals of patients with Hepatitis B surface antigen (HBsAg) and HCV antibody positivity to hepatologists. In South Africa and other low-middle income countries (LMICs), shortages of specialized medical professionals emphasize the need to train all healthcare professionals and patients on viral hepatitis and the implication of notifications to improve linkage to care. At the time of the study, South African guidelines with clear algorithms for viral hepatitis testing and referral systems were in preparation.

Our study was limited as it was conducted in only two hospitals and in only one province. The findings do not represent the entire national public health care sector nor the private hospitals. However, Gauteng is the most populated province in South Africa and the two hospitals are large referral hospitals. Therefore, the results do provide a significant baseline in assessing the knowledge of the health care professionals with regard to the current hepatitis notification system in South Africa. As the notification system in South Africa is being updated to an electronic platform, it should become quicker and easier to notify. However, training and awareness is imperative for any notification system (electronic or paper-based) to work efficiently. The other limitation of the study is that this was self-reported information, which could introduce information bias.

## Conclusion

As shown in other countries, our study suggested that the overall knowledge of notification of viral hepatitis is generally poor among health care professionals. The majority of the health care professionals reported that they had not received adequate training on notifiable medical conditions. Therefore, training is recommended to improve the reporting rate of notifiable diseases. The notification form should also be simplified since some of the health care workers found it to be complicated. Training on notifiable medical conditions should include: legal requirements and penalties, policies/regulations and guidelines, clear case definitions, easy-to-follow flowchart on the notification process, visible list of all notifiable medical conditions, visibility and accessibility of notification forms (paper-based or electronic) and encourage referrals of positive cases. Providing feedback on reported cases to the health care professionals will encourage them to notify. Communication between the national notification system and laboratories should improve and strengthen surveillance of diseases. The limitation of the study is that this was self-reported information, which could introduce information bias.

## Additional files

Additional file 1: **Table S1.** Comparison of knowledge, attitudes and practices regarding notification of viral hepatitis between nurses and doctors, Gauteng province, South Africa, 2015: % reflect correct/positive answers. (DOCX 16 kb)

Additional file 2: **Figure S1.** Health care professional’s responses to practices on notification. Absolute numbers reported on bars, Gauteng province, South Africa 2015. **Figure S2.** Health care professional’s major reasons for not notifying. Absolute numbers reported on bars, Gauteng province, South Africa 2015. (DOCX 43 kb)

## Abbreviations

HAV: Hepatitis A virus
HBsAg: Hepatitis B surface Antigen
HBV: Hepatitis B virus
HCC: Hepatocellular Carcinoma
HCV: Hepatitis C virus
NDOH: National Department of Health
NICD: National Institute for Communicable Diseases
SA: South Africa
