# Supplementary material for: Exploring the knowledge, attitudes and practices (KAP) of health care professionals on viral hepatitis notification in Gauteng, South Africa, 2015
Source: Arch Public Health. 2018 Dec 6;76:75. doi: 10.1186/s13690-018-0319-8 (PMC6282396; doi:10.1186/s13690-018-0319-8)
Supplement: Supplementary file 1 — Table S1. Comparison of knowledge, attitudes and practices regarding notification of viral hepatitis between nurses and doctors, Gauteng province, South Africa, 2015: % reflect correct/positive answers. (DOCX 16 kb) [file 13690_2018_319_MOESM1_ESM.docx]

ICP: Infection control personnel
