# Supplementary material for: Exploring the knowledge, attitudes and practices (KAP) of health care professionals on viral hepatitis notification in Gauteng, South Africa, 2015
Source: Arch Public Health. 2018 Dec 6;76:75. doi: 10.1186/s13690-018-0319-8 (PMC6282396; doi:10.1186/s13690-018-0319-8)
Supplement: Supplementary file 2 [file 13690_2018_319_MOESM2_ESM.docx]

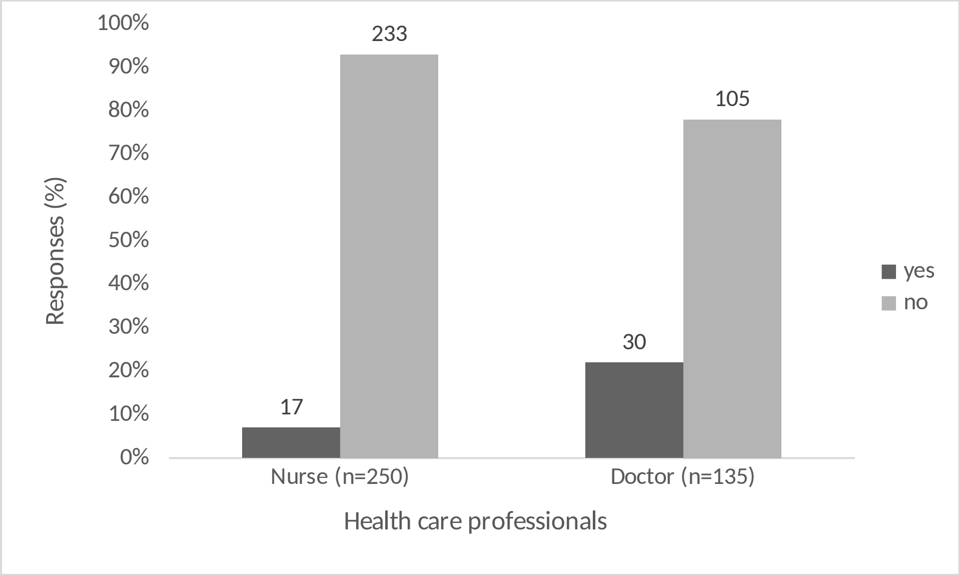
100%
90%
80%
70%
60%
50%
40%
30%
20%
10%
0%
Responses (%)
233
105
30
17
yes
no
Nurse (n=250)
Doctor (n=135)
Health care professionals
